# Supplementary material for: Integrating Metabolomics and Network Pharmacology to Explore the Mechanism of Xiao-Yao-San in the Treatment of Inflammatory Response in CUMS Mice
Source: Pharmaceuticals (Basel). 2023 Nov 14;16(11):1607. doi: 10.3390/ph16111607 (PMC10675308; doi:10.3390/ph16111607)
Supplement: Supplementary file 1 [file pharmaceuticals-16-01607-s001.zip › Figure S1 Multivariate statistical analysis of metabolic characters of mouse splenic samples.pdf]

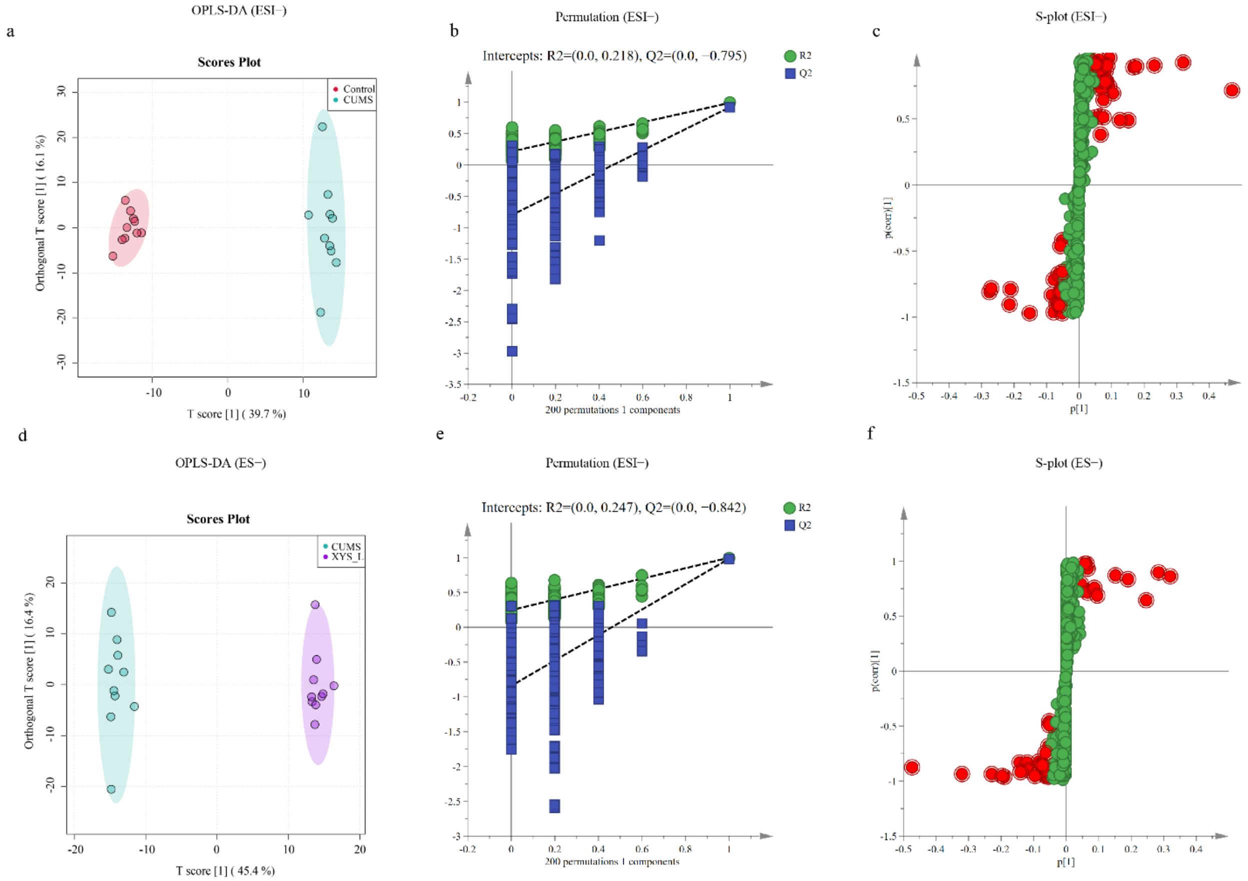

Figure S1 The OPLS-DA score graph**(a)**, permutation**(b)** and S-plot**(c)** of CUMS vs. XYS-H groups as well as the plot of OPLS-DA**(d)** permutation**(e)** and S-plot**(f)** of CUMS vs. XYS-H for negative ion mode.
